# Fatal accident inquiries into 83 deaths in Scottish prison custody: 2010–2013

**DOI:** 10.1192/bjo.2020.121

**Published:** 2020-10-30

**Authors:** Sheila M. Bird

**Affiliations:** Cambridge University's MRC Biostatistics Unit, UK; and Edinburgh University's College of Medicine and Veterinary Medicine, UK

**Keywords:** Prisoner deaths, fatal accident inquiries, waiting times, written determinations, pharmacovigilance

## Abstract

**Background:**

The only non-legal reference in Lord Cullen's Review of fatal accident inquiry (FAI) Legislation in Scotland (2009) was my audit of FAIs into 97 deaths in prison custody in Scotland, 1999–2003: recommending that waiting time from prisoner death to end of FAI should be less than 1 year for 90% of FAIs, and epidemiological rules for FAIs to have a written determination versus formal findings.

**Aims:**

Audit of FAIs into 83 deaths in Scottish prison custody in the period 2010–2013.

**Method:**

Assessement of waiting times from prisoner death to end of FAI; dissemination of written determinations; self-inflicted death rate per 1000 prisoner-years; cause of natural deaths; and yellow card submissions. Detailed cross-checking was nec37essary between Scottish Prison Service and courts’ websites and the Scottish Fatalities Investigation Unit.

**Results:**

Of 83 FAIs into deaths in Scottish prison custody, 2010–2013, 37 (45%) were long-awaited (ongoing >2 years after the prisoner's death); 16 (19%, 95% CI 11–28%) beyond 3 years. Of 37 long-awaited FAIs, 27 made written determinations but only 12 of these (44%) were published. Self-inflicted deaths numbered 36: 1.1 per 1000 prisoner-years (95% CI 0.75–1.48). Of 47 deaths from natural causes, cardiovascular disease accounted for 23 (49%, 95% CI 34–63%); liver disease was implicated in 10 of 47. To support pharmacovigilance, submissions were made to Medicines and Healthcare Regulatory Agency for eight deaths (10%, 95% CI 4–19%).

**Conclusions:**

FAIs into prisoner deaths in Scotland are too long-awaited given that four (5%) identified precautions that could have prevented death.

## Audit of fatal accidental inquiries into deaths in Scottish prison custody

A fatal accident inquiry (FAI) is conducted by a sheriff or more senior judge and is mandatory for a death in Scottish prison custody. Such deaths constitute half of Scotland's mandatory FAIs and a third of all FAIs.^1^ In my audit of 97 FAIs into deaths in 1999–2003 in Scottish prison custody found 54 were self-inflicted (defined as suicide or undetermined intent); 63% of self-inflicted deaths occurred on remand (95% CI 50–77%) versus 5 of 43 deaths from other causes.^2^ I recommended that the waiting time from prisoner death to end of FAI should be less than 1 year for 90% of FAIs; and proposed three epidemiological rules for identifying which FAIs merited a written determination to be posted on the Scottish Courts and Tribunal Service (SCTS) website for others to learn from. For example, deaths from natural causes for prisoners on remand (that is: untried) are exceptional epidemiologically. I also identified two prisoner deaths in which the same drug toxicity was implicated, so that yellow card notifications to the UK's Medicines and Healthcare products Regulatory Agency (https://yellowcard.mhra.gov.uk/) would have been warranted.

## Review of FAI egislation in Scotland by Lord Cullen

Lord Cullen retired as Lord President of the Court of Session in November 2005 having conducted the Public Inquiries into the Piper Alpha disaster and into the shootings at Dunblane Primary School and chaired the Ladbroke Grove Rail Inquiry. In 2008, he was appointed by Scottish Ministers to review the operation of the Fatal Accidents and Sudden Deaths Inquiry (Scotland) Act 1976 that governed the system of judicial investigation of sudden or unexplained deaths in Scotland so as to ensure that Scotland had an effective and practical system of public inquiry into deaths that was fit for the 21st century.

Most of Lord Cullen's recommendations were accepted by the Scottish Government, and shaped the Inquiries into Fatal Accidents and Sudden Deaths etc. (Scotland) Act 2016, which received Royal Assent on 14 January 2016.

Lord Cullen recommended that there should be a central FAI team ‘*to track cases*, *ensure adequate resources for investigations, give guidance in the light of previous FAIs*,’ and ‘*to maintain statistics relating to the different types of case, their progress and timing, thereby ensuring that preparation proceeded as expeditiously as possible*’ (recommendations 12–15).^1^

Moreover,

‘*sheriffs should use a standard form of determination, incorporating, according to the nature of the case, findings in fact, findings related to Section 6 (1) of the Fatal Accidents and Sudden Deaths Inquiry (Scotland) Act 1976, a note on the evidence and issues, and such recommendations, if any, as she or he considers appropriate. Subject to redaction as appropriate*, *the Scottish Courts and Tribunals Service website should contain all determinations; and be searchable*.’^1^

For FAIs, see Scottish Courts and Tribunal Service website.^3^ When a recommendation is made by a sheriff, the entity or body to whom it is directed should be under a duty to make a written response within a period set by the sheriff, stating whether and to what extent it has implemented, or intends to implement, the recommendation, or – if not – for what reason or reasons; there should be an annual report to the Scottish Parliament. In addition, ‘the sheriff should have power to direct to whom a copy of the determination should be sent for the dissemination of the lessons of the FAI’ (recommendations 28–34).^1^

## Suicides in prison custody: historical context and risk factors

Historically, rates of suicide in Scotland, and in Scottish prison custody, have been higher than the corresponding rates in England and Wales.^4–6^

At the turn of the 21st century, male prisoners’ suicide rate was four to five times higher than the age-appropriate national suicide rate not only in the UK^5,7^ but also in Germany^8^ (standardised suicide rate in 2000–2013 of 4.3, 95% CI 3.9–4.7); France^9^ (male suicide rate in 2006–2009 of 1.8 per 1000 prisoner-years, 95% CI 1.6–2.0); and in New South Wales, Australia^10,11^ where males’ suicide rate in prison was 1.3 per 1000 prisoner-years (95% CI 1.0–1.5) in 1988–2002. In the USA, suicides accounted for one-third of local jail inmate deaths (0.44 per 1000 male local jail inmates, 2010–2013)^12^ but for only 6% of inmate deaths in state prisons.^13^

The self-inflicted death rate in prison custody in England and Wales^14–17^ increased from 0.7 per 1000 prisoner-years in 2010–2012 (95% CI 0.59–0.79, 177 self-inflicted deaths, 74 on remand, average prison population 85 475) to 1.0 per 1000 prisoner-years in 2013–2015 (95% CI 0.87–1.12, 255 self-inflicted deaths, 91 on remand, average prison population 85 181). Her Majesty's Chief Inspector of Prisons for England and Wales^18^ questioned whether recommendations by the Prisons and Probation Ombudsman to prevent suicides had been implemented sufficiently well.

Risk factors for suicide in prison include: young age (under 21 years^6^); first-time prisoner; being on remand;^9,19^ period soon after reception into prison (first 24 h, first week, first month^19–21^); opioid dependence,^4,6,21–23^ which is highly prevalent; alcohol dependence; mental health problems including self-harm;^20,24^ suicidal ideation or risk behaviours while in the custody of police, court or prisoner escort before being received into prison; conviction for murder, rape or other serious sexual offence, or violent offending;^9^ long or unexpectedly long sentence;^25^ other trouble and strife – marital disharmony, worries about a partner's fidelity or children; being at risk from, or in debt to, other prisoners; concern expressed by family member or friend. There is strong evidence that uncontrolled symptoms of opiate withdrawal have played a part in prisoners’ suicides, especially soon after reception.^21–23^

## Scottish Prison Service: pertinent change since 2003, the end date for previous audit

The Scottish Prison Service (SPS) adopted opioid substitution therapy as the healthcare standard from 2003;^25^ random mandatory drugs testing of prisoners ended; and revisions of the SPS's suicide prevention policy, previously known as Act 2 Care, have been made. Scottish prisons engaged with Scotland's Hepatitis C Virus (HCV) Action Plans^26–28^ to promote HCV testing of older, former injecting drug users, a high proportion of whom were likely to be HCV carriers.^29^ In addition, Scottish prisons offer HCV testing universally to current injectors together with antiviral treatment for all HCV carriers.

In January 2011, Scotland became the first country with a national naloxone programme that was evaluated over 5 years; included Scotland's prisons; and demonstrated 50% reduction (95% CI 39–60%) in the proportion of Scotland's opioid-related deaths with a 4-week antecedent of prison release.^30–33^ Also, in 2011, legislation came into effect in Scotland that discouraged custodial sentences of less than 3 months.^31^ In November 2011, Scotland's National Health Service became responsible for prisoners’ health. Scottish prisons no longer have in-patient beds, nursing cover begins at 07.00 h and ends at 21.30 h on weekdays (shorter hours at weekends) but there is on-call doctor cover overnight.

## Aims of the present audit

The legislative framework for FAIs into deaths in Scottish prison custody in 2010–2013 remains the Fatal Accidents and Sudden Deaths Inquiry (Scotland) Act 1976 and the Fatal Accidents and Sudden Deaths Procedure (Scotland) Rules 1977. In auditing these 83 FAIs, this paper aims to:

- document the waiting times from prisoner death to end of FAI, compared with 1999–2003;
- estimate the suicide (and self-inflicted death) rate per 1000 prisoner-years in Scottish prison custody;
- comment on the cause of natural deaths in the prisoner population, compared with 1999–2003;
- highlight lessons to be learned from Section 6.1.(c), (d) or (e) findings in the context of long waiting times.

Of 97 deaths in Scottish prison custody in 1999–2003, 54 were self-inflicted, and only one FAI took longer than 3 years to conclude.^2^ My three epidemiological rules for FAIs that should have a written determination posted on the SCTS website were validated for deaths in Scottish prison custody in 1994–1998,^2^ and are as follows.

- Rule 1: death by natural causes of prisoner on remand.
- Rule 2: younger than 21 years of age at death, death self-inflicted by means other than hanging, or cause of death involves drug toxicities.
- Rule 3: FAI in progress more than 1 year after prisoner death.

## Method

Three data sources on FAIs into prisoner deaths were accessed.

First, the SPS website (see ^34^), which tabulates prisoner deaths by the calendar year of death, names the establishment where the prisoner was detained (Her Majesty's Prison (HMP) or young offender institute) and gives: name of the deceased, date of admission, date of death; also the deceased prisoner's age in completed years, gender, ethnic group and legal status (as remand or convicted); and identifies the cause of death as undetermined intent/overdose, natural causes or suicide.

The second data source was the SCTS website, but many FAIs for prisoner deaths in 2010–2013 could not be located on the SCTS website.^3^ Hence, recourse was necessary to a third data-source, the further detail held by SPS about FAIs: the date that FAI concluded (the written determination date is typically later than the FAI end date); on the progress of FAIs; on the cause of death as determined by FAI; and on whether the FAI made findings at Section 6.1.(c), (d) or (e) of the Fatal Accidents and Sudden Deaths Inquiry (Scotland) Act 1976. SPS's further information helped by identifying FAIs with apparently long waiting times, at least 730 days, from date of prisoner death to FAI end date according to SPS, see Supplementary Appendix available at https://doi.org/10.1192/bjo.2020.121.

To minimise requests to staff at the Crown Office and Procurator Fiscal Service for locating and copying to me those FAIs that were missing from the SCTS website, requests were made only if: (a) waiting time from prisoner death to FAI end (according to SPS) exceeded 730 days; (b) the prisoner's death qualified as exceptional by Rule 2.

In addition, Scotland's published information by age group on the number of prisoners held in Scottish prison custody at 30 June^35^ was used to approximate the age-specific suicide rates (also self-inflicted death-rates) in 2010–2013 per 1000 prisoner-years; and to derive 95% confidence intervals.

Life table methods were used to estimate, and stem and leaf diagrams to visualise, the waiting time from prisoner death to FAI end for all prisoner deaths in 2010–2013, including when differentiated by natural causes versus undetermined intent/overdose or suicide. In 1999–2003, deaths by natural causes were subdivided to highlight cardiovascular deaths. Here, I also focus on the subset of deaths in which liver disease was implicated (other than as a site of metastasis).

## Results

Table 1, a stem and leaf diagram, shows the waiting time from 83 prisoner deaths in 2010–2013 to end of FAI: bold identifies the waiting times for 36 prisoners whose death was by suicide (1 by cocaine toxicity) or an event of undetermined intent/overdose (4 by intoxication or toxicity; one whose cause of death could not be ascertained).

**Table 1:** Stem and leaf diagram for 83 waiting times from prisoner death to end of fatal accident inquiry^a^

	Leaf for waiting time: in the range 00–99 days		
	All deaths	Suicide or event of undetermined intent/overdose	Natural causes
Stem in 100s for waiting time, days			
1	33, 63, 68		33, 63, 68
2	76, 92, 13, 07, 12, 53, 77		07, 12, 13, 53, 76, 77, 92
3	**14**, **15**, 42, 00, 28, 84, **53**, 04, 80, **23**, **87**	**14**, **15**, **23**, **53**, **87**	00, 04, 28, 42, 80, 84
4	97, **96**, 70, 46, 99, 17, ***09***, 50, 21, **93**	***09***, **93**, **96**	17, 21, 46, 50, 70, 97, 99
5	53, **65**, **56**, 86, 47, 15, 31	**56**, **65**	15, 31, 47, 53, 86
6	**71**, 86, **69**, 08, 48, **30**, **81**	**30**, **69**, **71**, **81**	08, 48, 86
7	**55**, 02, 85, 65, 65, **97**	**55**, **97**	02, 65, 65, 85
8	***03***, 89, 02, 33, **15**, **09**	***03***, **09**, **15**	02, 33, 89
9	**90**, 31, **62**, 29, **33**	**33**, **62**, **90**	29, 31
10	55, **98**, **50**, **71**, ***20***, 95, ***97***	***20***, **50**, **71**, ***97***, **98**	55, 95
11	51, **64**, ***04***, 30, 52, **17**	***04***, **17**, **64**	30, 51, 52
12	**05**, **71**, 46, **51**	**05**, **51**, **71**	46
13	**25**, 23+	**25**	23+
14	**11**, **28**	**11**, **28**	
Totals deaths, *n*	83	36	47
Median, days	669	812	515
Quartiles, days	384–990	556–1104	304–785

a. Bold is used for deaths by suicide; italic used for deaths by suicide or event of undermined intent/overdose.

Median waiting time to end of FAI was 669 days, or 22 months; 37 FAIs took longer than 2 years (45%, 95% CI 34–55%) with 16/83 continuing beyond 3 years (19%, 95% CI 11–28%). Median waiting time was longer than 2 years for deaths by suicide or event of undetermined intent/overdose (812 days) but less than 1.5 years for deaths from natural causes (515 days).

Of the 36 deaths by suicide or event of undetermined intent/overdose, only four FAIs ended within 1 year, 22 lasted for longer than 2 years (61%, 95% CI 45–77%) with 11/36 continuing beyond 3 years (31%, 95% CI 16–46%). The corresponding statistics for the 47 deaths from natural causes were: 14 ended within 1 year, 15 lasted for at least 2 years (32%, 95% CI 19–45%) with 5 continuing beyond 3 years (11%, 95% CI 2–19%).

Table 2 shows that the age distribution for deceased prisoners was different by cause of death: mean age at death by suicide or undetermined intent/overdose was 32.9 years (s.e. = 1.7), considerably younger than for deaths by natural causes: 53.5 years (s.e. = 1.9). The youngest person who died by suicide was 18 years old, for whom, surprisingly because contrary to Rule 2, only formal findings (constitute name of deceased, date of birth, date and place of death, cause of death) were made despite a waiting time from death to inquest verdict of 1271 days. All nine deaths under 25 years of age were self-inflicted, seven of them on remand.

**Table 2:** Stem and leaf diagram for the age at death of 83 prisoner deaths by cause of death^a^

	Leaf for prisoner's age at death: units in the range 0–9 years		
	Suicide^b^ or event of undetermined intent/overdose^c^	Natural causes, including those with mention of cardiovascular disease	Natural causes with any mention of liver disease
Stem in decades for age, years			
1	**8, 9^d^**, **9^d^, 9**		
2	***1^d^***, **2^d^**, **4^d^**, **4^d^**, **4^d^**, ***5^d^***, **5**, **6**, **7^d^, 7**, ***8***, **9**		
3	**0^d^, 1, 2**, **2**, **3^d^**, ***5***, **5^d^**, **6^d^**	1, 5^e^, 6, 8	1, 6
4	**0**, **0**, **1**, **1^d^**, **2^d^**, **3^d^**, **5^d^**, **6^d^**, ***7***, **7^d^**	0, 0, 1, 2^e^, 2, 2, 3, 4^e^, 4^e^, 4, 5^e^, 6^e^, 6, 7^e^, 7, 7, 8, 8	0, 1, 4, 6, 6
5	**5**, **6**	0^e^, 0^e^, 2, 3, 3^e^, 6^e^, 6^e^, 9^e^	3, 3
6		0^e^, 0^e^, 0^e^, 0, 1^e^, 1^e^, 1, 2, 5, 6^e^	0
7		2^e^, 3, 4^e^, 6, 8, 9^e^	
8		1^e^	
Total, *n*	36	47 (CVD: 23)	10
Median, years	31.5	50 (CVD: 56)	45
Quartiles, years	24–41	44–61 (CVD 46–61)	38–53
Age, mean (s.d.)	32.9 (10.4)	53.5 (12.8) (CVD 56.6 (12.1))	45 (8.7)

a. Bold is used for deaths by suicide; italic used for deaths by suicide or event of undermined intent/overdose.

b. Suicide: 17 remand, 14 convicted.

c. Undetermined intent/overdose: two remand, three convicted.

d. Remand.

e. With mention of cardiovascular disease.

Prisoners on remand accounted for 19/36 self-inflicted deaths (53%, 95% CI 36–69%); but for only 5 out of 47 deaths by natural causes, see Rule 1 and Supplementary Appendix.

The previous audit highlighted that 44% of 43 prisoner deaths from natural causes in 1999–2003 mentioned cardiovascular disease.^2^ The proportion in 2010–2013 was similar at 49% (23/47, 95% CI 34–63%).

Additionally, in the current audit, liver disease (excluding metastasis to liver) was mentioned in ten deaths from natural causes at a mean age of 45 years (s.e. = 2.7). The ten included a prisoner (J.A.C. – see Supplementary Appendix for further details) who died at 60 years of age in August 2013, having apparently been HCV infected during his incarceration in England in 1997 before being transferred to, and receiving HCV treatment in, Scottish prison custody. End-stage liver disease was also the cause of an eleventh death: that of 35-year-old prisoner (M.M. – see Supplementary Appendix) in 2011, who was released on licence from HMP Barlinnie on his day of death, for whom there was no FAI.

Table 3 shows the age distribution of those held in Scottish prison custody on 30 June, 2010 to 2013, from which I estimate that the death rate per 1000 prisoner-years in Scottish prison custody was 1.2 under 40 years of age (95% CI 0.7–1.6), 5.0 at ages 40–59 years (95% CI 3.4–6.6) and 22 per 1000 prisoner-years at ≥60 years of age (95% CI 12–33). The suicide rate in Scottish prison custody in 2010–2013 was approximately 1.0 per 1000 prisoner-years (95% CI 0.62–1.30). Of the 31 suicides, 17 were prisoners on remand. The self-inflicted death rate was 1.1 per 1000 prisoner-years (95% CI 0.75–1.48).

**Table 3:** Age distribution for deceased prisoners and for those held in Scottish prison custody (on 30 June) in 2010–2013^a^

Age of prisoners, years	Suicides by prisoners in 2010–2013 (approximate rate per 1000 prison-years)	Deceased prisoners in 2010–2013 (approximate rate per 1000 prison-years)	Held in Scottish prison custody on 30 June in 2010–2013				
			2010	2011	2012	2013	4-year mean
<21	4 (1.3)	4 (1.3)	949	819	747	587	776
21–29	9 (0.8)	12 (1.1)	2848	2889	2873	2643	2813
30–39	7 (0.7)	12 (1.3)	2204	2330	2462	2472	2367
40–49	9 (1.6)	28 (5.0)	1353	1408	1461	1397	1405
50–59	2 (1.0)	10 (4.9)	445	483	527	568	506
≥60	0 (0)	17 (22.3)	184	177	185	216	191
Totals	31 (1.0)	83	7983	8106	8255	7883	8057

a. Test for homogeneity for in prison suicide rate by age group, according to which the expected counts are 2.99; 10.82; 9.11; 5.41; 2.68 for >50 years, leading to chi-squared on 4 d.f. of 3.70.

Of 37 FAIs with a waiting time from prisoner death to end of FAI of at least 2 years, one was ongoing still at 17 June 2017 with written determinations awaited from two others – all three had reported by 24 September 2017, see Supplementary Appendix. As shown in Table 4, these 37 long-awaited FAIs resulted in 27 written determinations, only 12 of which were posted on SCTS's website by 24 September 2017 (44%; 95% CI 26–63%).

**Table 4:** Availability by 17 September 2017 on the website of Scottish Courts and Tribunals of fatal accident inquiries into deaths in Scottish prison custody in 2010–2013 with waiting times from prisoner death to inquest verdict of more than 2 years

Year, available on website	Death by suicide, *n*		Death by undetermined intent/overdose, *n*		Death by natural causes, *n*		Total, *n*
	Written determination	Formal findings	Written determination	Formal findings	Written determination	Formal findings	
2010						
Yes	3				1		4
No		2					2
2011							
Yes	2				1		3
No	2		2		2	2	8
2012							
Yes			1				1
No	4	2			2	4	12
2013							
Yes	1		1		2		4
No	2				1		3
Total for 2010–2013							
Yes	6		2		4		12
No	8	4	2		5	6	25^a^

a. Includes ten formal findings.

Application of Rules 1 and 2 identified 19 FAIs as epidemiologically exceptional prisoner deaths in 2010–2013. Rule 3, originally stated as FAI into prisoner's death still in progress at 1 year after the date of death, has been updated to 3 years, thereby identifying 16 cases or one-fifth, see Table 1. Whereas a fifth of 97 FAIs into prisoner deaths in 1999–2003 were still in progress at 1 year after the prisoner's date of death, for deaths in Scottish prison custody in 2010–2013, Rule 3 – without updating beyond 1 year (65/83, 78%) or 2 years (37/83, 45%) – would have captured a substantially higher proportion of FAIs into deaths in Scottish prison custody than in 1999–2003, as evident from Table 1.

Six of the 16 FAIs that met Rule 3 (updated to 3 years) overlap with the 19 cases identified by Rules 1 and 2, namely: A.R., S.J., R.McC., S.M., F.C. and J.J. (see Supplementary Appendix). Hence, Rules 1 to 3 (updated to 3 years) identified 29 out of 83 FAIs into deaths in Scottish prison custody in 2010–2013 that should clearly have been posted on the SCTS website because they are exceptional in epidemiological terms: only 15 of these 29 selected FAIs (52%, 95% CI 33–70%) were posted by 24 September 2017.

By contrast, of seven FAIs into prisoner deaths in 2010–2013 which, according to the SPS, made Section 6.1 (c), (d) or (e) findings (see Supplementary Appendix) only one (N.McN.) was not posted on the SCTS website by 24 September 2017.

Waiting times in days from prisoner death to end of FAI + wait for written determinations and Section 6.1 findings for these seven FAIs were: 669 + 43 days (Section 6.1.(c)); 931 + 0 days (6.1.(e), not posted); 1071 + 86 days (6.1.(e)); 1097 + 302 days (6.1.(c) and (e)); 1130 + 88 days (6.1.(e)); 1399 + 48 days (6.1.(c)); and 1428 + 35 days (6.1.(c) and (e)). Briefly, the four sets of reasonable precautions (Section 6.1.(c)) whereby death might have been avoided were as follows:

- for named personnel to have ensured that prisoner in segregation unit was assessed by doctor as soon as practicable and as often as necessary thereafter but at least once a week;
- for named prison officer to have placed a copy of alerting letter (from depute procurator fiscal to governor) in the prisoner's medical records and attached a copy also to the prisoner's Act2Care booklet;
- had the deceased avoided taking non-prescribed drugs illegally and in lethal quantities and, when asked, told the nurse what he had taken; had prison officers conducting hourly observations had information about adverse drug effects and how symptoms might exhibit themselves; had prison officers undertaking hourly observations ensured that they obtained both a visual and a coherent verbal response to confirm that the prisoner was oriented to time, place and identity; had named prison officer, having determined that the prisoner was not so oriented, sought a further medical assessment for the prisoner;
- had automated external defibrillators (AEDs) been readily accessible for use by prison officers on night shift; and had prison officers on night shift been given training and instruction on when and how to use AED.

There was no indication that the UK's Medicines and Healthcare products Regulatory Agency had been informed about any deaths in which drug toxicity or polypharmacy was implicated and so yellow card notifications (https://yellowcard.mhra.gov.uk/) were made by me for the eight deaths discussed below (10%, 95% CI 4–19%), none of which had been previously reported.

HMP Barlinnie's dihydrocodeine and/or benzodiazepine detoxification programmes featured in two deaths of undetermined intent/overdose (A.R. and R.J.D.), the one described explicitly as dihydrocodeine intoxication, the other as cause unascertained (see Supplementary Appendix). Toxicity from prescribed methadone in older prisoners (D.J.G., aged 47 and W.C., aged 46 years), without or with co-presence of amitriptyline and gabapentin (inter alia), may have had a role in two deaths from cardiovascular causes.

Four other deaths in which drug toxicity was implicated were those of K.F. (aged 47 years, coronary atherosclerosis and nefopam toxicity); P.W.G. (aged 35 years, amitriptyline and chlorpheniramine intoxication); F.C. (aged 42 years, multidrug toxicity: supervised methadone at 60 mg per day; F.C.'s other prescribed medications, which included amitriptyline at 50 mg three times a day, were dispensed weekly; also present at autopsy was an unquantifiable level of quetiapine, source unknown); and J.J. (aged 28 years, who overdosed having consumed lethal quantities of buprenorphine, gabapentin and phenazepam, please see Section 6.1.c findings for J.J at (c) above).

## Discussion

### Timeliness

Rules 1–3 (updated to 3 years) highlighted a similar proportion of FAIs for enhanced scrutiny in 2010–2013 (35%) as in 1999–2003 (38/97 or 39%). Updating to 3 years was necessary because, whereas in 1999–2003 only a fifth of FAIs continued beyond 1 year, waiting times from prisoner death to end of a FAI had increased so unacceptably that a fifth of FAIs into prisoner deaths in 2010–2013 continued beyond 3 years (16/83) and the median waiting time was 22 months.

Performance measures^36^ that heed only the delay from death date to FAI start omit the often protracted interval from FAI start to FAI end. The further wait from FAI end to written determination can exceed 90 days^2^ and did so for one of seven written determinations that made Section 6.1.(c), (d) or (e) findings, the wait from FAI end to written determination being an extraordinary 302 days, see Supplementary Appendix.

In 1999–2003, only 5 out of 42 written determinations were posted on the SCTS website. Table 4 showed that 27 of the 37 FAIs that continued 2 years after the prisoner's death made written determinations, 12 of which (44%, 95% CI 26–63%) were posted on the SCTS website, a welcome improvement on 1999–2003 but far short of 100% compliance.^1,2^

### Causes of death

In 1999–2003, 19/43 (44%) deaths from natural causes involved cardiovascular disease as did 23/47 (49%) in 2010–2013; see also^37^. Particularly noteworthy in 2010–2013 is that liver disease (excluding liver metastasis) was implicated in ten deaths from natural causes at a mean age at death of only 45 years (s.d. = 9 years). End-stage liver disease was also the cause of the death of a prisoner who was released on licence from HMP Barlinnie on the day of his death. The youngest age of the liver-rated deaths was from acute liver failure at 31 years of age: his probable HCV carriage had not been diagnosed. In the written determinations for 36-year-old R.W.M., the sheriff asked what the National Health Service's (and SPS's) policy was on the discharge of prisoner–patients in need of nursing care; and remarked that either greater provision for nursing care in prisons or consideration of compassionate release would be needed in view of the likely number of prisoners with liver disease, which this audit endorses.

The proportion of deaths in Scottish prison custody that were self-inflicted reduced from 54/97 (56%) in 1999–2003^2^ to 36/83 (43%) in 2010–2013 (*P* = 0.10). The self-inflicted death rate was 1.1 (95% CI 0.75–1.48) per 1000 prisoner-years, higher than for prisoners in England and Wales in 2010–2012, but consistent with the provisional rate in England and Wales of 1.0 (95% CI 0.87–1.12) per 1000 prisoner-years in 2013–2015.

Four FAIs (5%) identified precautions that might have prevented death, as detailed in the Results section.

### Yellow card submissions

Eight yellow card submissions (10%) affirm the potential importance of mandatory FAIs for deaths in Scottish prison custody for UK pharmacovigilance. One of the eight FAIs (for J.J.) made Section 6.1.(c) findings on precautions that might have prevented his death. Gao et al^38,39^ highlighted the sharp increase with age in methadone-specific deaths among Scotland's methadone clients and association with circulatory comorbidity, which is pertinent for two yellow card submissions. Two others concerned detoxification programmes that are not recommended in UK guidance; and, contrary to the sheriff's view, neither UK's 2006 guidelines for the management of opioid dependency nor those in 2017^40^ recommended the prison's choice of the dihydrocodeine detoxification programme.

A Freedom of Information request that I made disclosed that, prior to 2018, the Prisons and Probation Ombudsman for England and Wales had made no yellow card submissions for any death in prison custody. Based on around 300 reports per annum, 30 yellow cards annually would have been warranted if similar prison-based practices applied in England and Wales as in Scotland. Coroners and sheriffs should consider whether the inquest or FAI at which they preside has identified a potentially serious adverse event that could relate to the prisoner's medications; if so, yellow card submission could assist pharmacovigilance. Moreover, UK's Medicines and Healthcare products Regulatory Agency could itself scrutinise written determinations and reports by the Prisons and Probation Ombudsman for drug alerts.

Indeed, coroners in England and Wales are obliged to make reports when they believe it is possible to prevent future deaths. Ferner et al^41^ examined 500 such coroners’ reports to identify 99 in which medicines or the medication process played a part: adverse reactions to prescribed medicines (*n* = 22), omission of necessary treatment (*n* = 21), failure to monitor treatment (*n* = 17) and poor systems (*n* = 17). Four safety warnings from the Medicines and Healthcare products Regulatory Agency that were based on coroners’ warnings^42^ – an alert to warning rate of 4% – seems a notable success-rate.

### Other reflections

Bad practices^2^ – such as failures by the Crown, police or SPS to secure evidence as for a crime scene, failure to allow specialist neuropathology for a sudden death in epilepsy, incorrect recording or conduct of hourly observations and overreliance on anecdotal rather than empirical evidence – were infrequent but not absent, see the Results section and Supplementary Appendix.

Deaths in prison custody are regretted by officers and fellow prisoners alike, who have often been united in their concern for, and efforts to save, the deceased. Not all deaths in prison can be prevented, but the SPS has been successful in reducing self-inflicted deaths, especially those by prisoners under 25 years of age.^5^ Deaths of older prisoners in Scottish prison custody are now under scrutiny, including in a thematic report on older prisoners by Her Majesty's Chief Inspector of Prisons for Scotland (2017).^42^ See also the thematic report on social care in prisons in England and Wales by HM Inspectorate of Prisons and the Care Quality Commission (2018);^43^ and on America's ageing prison population.^37^

Scotland's long delays from prisoner death to end of FAI persist:^44^ The SPS website records 105 deaths in Scottish prison custody in 2014–2017, but 10 out of 29 prisoner deaths in 2017 were awaiting determination at 1 November 2019 as were 4 out of 28 prisoner deaths in 2016 (all 4 had waits in excess of 3 years). Hence, for prisoner deaths in 2019, the SPS website now provides the prisoner's provisional medically certificated cause of death ahead of the FAI.

### Limitations

First of several limitations to the present study is reliance on one person's abstraction from sheriffs’ written determinations, themselves idiosyncratic in style^1^ – unlike reports by Prisons and Probation Ombudsman.

Methodology for the 2010–2013 audit differs from its predecessor in 1999–2003^2^ although the SPS's list of prisoner deaths was the starting point for both. Whereas Freedom of Information requests underpinned the 1999–2003 audit, detailed cross-checking between the SPS, SCTS website and the Scottish Fatalities Investigation Unit was relied upon in 2010–2013 – but only in respect of FAIs that lasted 2 years or more or met Rule 2. Finally, SPS's use of the term formal findings may have indicated that no Section 6.1.(c), (d) or (e) finding was made rather than that there had been no written determination. For FAIs that ended within 2 years of the prisoner's date of death and that did not meet Rule 2, the SPS's use of formal findings was not cross-checked.

### Recommendations

(a) The major recommendation remains that waiting time from prisoner death to end of FAI should be less than 1 year for 90% of FAIs.^1^ Performance measures that heed only the delay from date of death to FAI start need to be redefined to measure the delay from date of death to FAI end.^44^ Families want some good to come of their tragedy. The waiting time that matters is from date of death to FAI end, not from date of death to FAI start.

(b) Yellow card submission by sheriffs or coroners could assist pharmacovigilance and reduce iatrogenic harm because of prescribing practices (in particular, detoxification regimes). And UK's Medicines and Healthcare products Regulatory Agency could itself scrutinise written determinations and reports by the Prisons and Probation Ombudsman for evidence about serious adverse drug reactions.

(c) Written determination is essential about the death of any child in legal custody.

(d) Waiting time for written determinations should be less than 90 days for 90% of written determinations; and all should be posted on the SCTS website.^1,2^

(e) For sheriffs’ advice to have an impact before a similar death occurs in the same or a different prison, there must be greater timeliness of FAIs than in 2010–2013 and comprehensive posting of written determinations (minimally redacted as necessary) on the SCTS website.^1^ The 2016 Act, in force from 15 June 2017, requires that determinations are published by the SCTS.

## Data Availability

Data availability is not applicable to this article as no new data were created or analysed in this study other than as set out in the tables provided.

## Supplementary material

For supplementary material accompanying this paper visit http://dx.doi.org/10.1192/bjo.2020.121. click here to view supplementary material
