# Supplementary material for: Fatal accident inquiries into 83 deaths in Scottish prison custody: 2010–2013
Source: BJPsych Open. 2020 Oct 30;6(6):e132. doi: 10.1192/bjo.2020.121 (PMC7745234; doi:10.1192/bjo.2020.121)
Supplement: Supplementary file 1 [file bjosup.zip › S2056472420001210sup001.doc]

**APPENDIX:** Deaths in Scottish prison custody, 2010-2013

| **Initials**,  SPS-status  (C = convicted, R = remand)  *HM Prison*  **[Section 6.1 c, d or e finding;]** | **Age** | **Dates** | | **Death** to **FAI-end**  *If > 730 days, was FAI on website of Scottish Courts & Tribunal @ 17/06^/^17?* | | **FFs according to Scottish Prison Service or**  **WD: date** | **Cause of death**  **UO: undetermined intent/overdose**  **NC: Natural causes**  **SU: Suicide** |
| --- | --- | --- | --- | --- | --- | --- | --- |
|  |  | Death | End of FAI according to SPS |  |  |  |  |
| **16 deaths in Scottish prison custody in 2010.**  **FIVE out of 16 Fatal Accident Inquiries (FAIs) into prisoner-deaths had time from prisoner-death to FAI-end > 1095 days.**  **In THREE out of 7 FAIs with time from prisoner-death to FAI-end > 730 days, the determination could not be located on Scottish Courts & Tribunals website, 17 June 2017.** | | | | | | | |
| **RH**, C  *HMP Edinburgh* | 40 | 29/04/20**10** | 03/11/20**11** | 553 | **WD**: 08/11/**11** | | **NC**: 1a multi-organ failure, 1b pneumococcal pneumonia, 2 chronic HIV & liver cirrhosis |
| **RHMcG**, C  *HMP Addiewell* | 41 | 15/11/20**10** | 26/03/20**12** | 497 | **WD:** 26/03/12 | | **NC**: epilepsy |
| **SH**, C  *HMP Cornton Vale* | 47 | 11/01/20**10** | 07/03/20**13** | 1151 *(Yes)* | **WD**: 03/04/**13** | | **NC**: 1a) complications of peritonitis & sepsis, 1b) metastatic colon cancer |
| **EB,** C  *HMP Perth* | 47 | 22/05/20**10** | 22/02/20**11** | 276 | ?? | | **NC**: septic shock secondary to pneumonia worsened by HIV&AIDS |
| **RWM,** C  *HMP Perth* | 36 | 03/12/20**10** | 21/09/20**11** | 292 | **WD:** 01/11/**11** | | **NC**: end-stage liver disease, cirrhosis of liver & HCV |
| **AWT**, C  *HMP Perth* | 61 | 12/12/20**10** | 13/07/20**11** | 213 | FF | | **NC**: atherosclerotic coronary artery disease |
| **SAP**, C  *HMP Barlinnie* | 27 | 02/04/20**10** | 17/12/20**12** | 990  ***(No)***  ***Barlinnie*** | FF | | **SU**: hanging |
| **SR**, C  *HMP Barlinnie* | 26 | 15/04/20**10** | 30/11/20**13** | 1325  *(Yes) joint-FAI* | **WD:** 31/01/**14** | | **SU**: hanging |
| **JB**, R  *HMP Barlinnie* | 27 | 13/08/20**10** | 30/11/20**13** | 1205  *(Yes)*  *joint-FAI* | **WD:** 31/01/**14** | | **SU**: hanging |
| **WJM**, C  *HMP Barlinnie* | 29 | 07/06/20**10** | 14/08/20**13** | 1164  *(Yes)* | **WD**: 15/11/**13** | | **SU**: hanging |
| **SS**, R  *HMP Barlinnie* | 43 | 28/09/20**10** | 30/07/20**12** | 671 | FF | | **SU**: hanging |
| **GRR**, R  *HMP Barlinnie* | 30 | 28/10/20**10** | 21/11/20**12** | 755  ***(No)***  ***Barlinnie*** | FF | | **SU**: hanging |
| **PS**, R  *HMP Edinburgh* | 42 | 15/07/20**10** | 25/05/20**11** | 314 | **WD**: 15/06/**11** | | **SU**: death due to cocaine toxicity |
| **PH**, C  *HMP Greenock* | 32 | 18/11/20**10** | 29/09/20**11** | 315 | **WD**:28/10/**11** | | **SU**: hanging |
| **JPM**, R  *HMP Kilmarnock* | 24 | 08/05/20**10** | 24/11/20**11** | 565 | **WD**:24/11/**11** | | **SU**: hanging |
| **AAS**, R  *HMYOI Polmont* | 19 | 25/12/20**10** | 04/05/20**12** | 496 | **WD**: 04/05/**12** | | **SU**: hanging (suspension by cloth ligature) |
| **23 deaths in Scottish prison custody in 2011: one was not subject to FAI.**  **THREE out of 22 Fatal Accident Inquiries (FAIs) into prisoner-deaths had time from prisoner-death to FAI-end > 1095 days.**  **In EIGHT out of 11 FAIs with time from prisoner-death to FAI-end > 730 days, the determination could not be located on Scottish Courts & Tribunals website, 17 June 2017.** | | | | | | | |
| **RJD**, R  *HMP Barlinnie* | 21 | 03/11/20**11** | 15/01/20**14** | 803  ***(No)***  ***Barlinnie*** | WD: 15/01/14 | | **UO**: dihydrocodeine intoxication |
| **AR,** R  *HMP Barlinnie* | 25 | 07/01/20**11** | 15/01/20**14** | 1104  ***(No)***  ***Barlinnie*** | WD: 15/04/14 | | **UO**: cause of death could not be ascertained. |
| **RM**, C  *HMP Peterhead* | 61 | 18/05/20**11** | 25/04/20**12** | 342 | FF | | **NC**: carcinoma of the lung |
| **MM**, C  *HMP Barlinnie* | 35 | xx/05/20**11** | ***Procurator Fiscal decided FAI was not necessary into this prisoner’s death because MM was released on licence from HMP Barlinnie on death-date (xx) in May 2011*** | | | | **NC: end-stage liver disease** |
| **JMR**, C  *HMP Dumfries* | 73 | 11/11/20**11** | 23/03/20**12** | 133 | FF | | **NC**: death due to cancer |
| **TCR**, R  *HMP Edinburgh* | 56 | 30/06/20**11** | 25/04/20**12** | 300 | FF | | **NC**: cardiomegaly, valvular heart disease (aortic & mitral) & coronary artery atherosclerosis |
| **MR**, R  *HMP Edinburgh* | 35 | 07/01/20**11** | 09/12/20**12** | 702 | ?? | | **NC**: myocardial infarction, coronary artery atherosclerosis (stented on 4 January 2011) |
| **NFMcN**, C  *HMP Glenochil*  **[Section 6.1.e]** | 72 | 31/07/20**11** | 16/02/20**14** | 931  ***(No)***  ***Glenochil*** | **WD**:16/10/**14** | | **NC**: metastatic lymphoma of colon, sepsis; secondary causes of atherosclerotic coronary vascular disease & hypertension. |
| **DJG**, C  *HMP Glenochil* | 47 | 03/03/20**11** | 17/01/20**13** | 686 | **WD**:17/01/**13** | | **NC**: methadone toxicity, coronary artery disease |
| **GJC**, C  *HMP Glenochil*  **[Section 6.1.e]** | 78 | 07/03/20**11** | 10/04/20**14** | 1130  *(Yes)* | **WD:** 07/07/**14** | | **NC**: intracerebellar haemorrhage, secondary to long-standing hypertension. Less than 24 hours before death, a metastatic non-small cell lung cancer was diagnosed which had spread to liver but said cancer did not materially contribute to cause of death – contrary to original autopsy. |
| **RU**, C  *HMP Inverness* | 66 | 07/11/20**11** | 01/06/20**12** | 207 | ?? | | **NC**: ischaemic heart disease |
| **SKM**, C  *HMP Kilmarnock* | 31 | 17/11/20**11** | 07/10/20**14** | 1055  ***(No)***  **Kilmarnock** | **WD:** 27/10/**14** | | **NC**: 1a) acute liver failure due to 1b) chest infection due to septicaemia, due to 1c) liver cirrhosis |
| **RBR**, C  *HMP Perth* | 60 | 06/10/20**11** | 18/01/20**13** | 470 | ?? | | **NC**: 1) small bowel perforation & peritonitis, 2) small bowel infarction, 3) superior mesenteric artery thrombosis & atherosclerotic cardiovascular disease |
| **DH**, C  *HMP Peterhead* | 79 | 02/06/20**11** | 25/04/20**12** | 328 | FF | | **NC**: leaking atheromatous abdominal aortic aneurysm & coronary artery disease |
| **JF**, C  *HMP Peterhead* | 59 | 08/01/20**11** | 29/03/20**12** | 446 | ?? | | **NC**: ischaemic heart disease |
| **HLK**, R  *HMP Barlinnie* | 46 | 11/07/20**11** | 16/12/20**13** | 889  ***(No)***  ***Barlinnie*** | FF | | **NC**: a) presumed sepsis due to b) chronic drug & alcohol abuse with HCV & cirrhosis. |
| **FMG**, C  *HMP Open Estate* | 56 | 07/10/20**11** | 17/12/20**13** | 802  ***(No)***  ***Open Estate*** | FF | | **NC**: atherosclerotic coronary artery disease |
| **AJMcE**, R  *HMP Barlinnie* | 46 | 22/05/20**11** | 28/11/20**12** | 556 | **WD:** 04/12/**12** | | **SU**: hanging |
| **SJ**, R  *HMP Barlinnie* | 47 | 29/01/20**11** | 31/01/20**14** | 1098  ***(No) Barlinnie*** | **WD:** 31/01/**14** | | **SU**: haemorrhage from self-inflicted wounds on left-side of neck  (on remand re sexual offences; twice pretended to two women to have had non-Hodgkins lymphoma) |
| **BFG**, R  *HMP Greenock* | 35 | 20/07/20**11** | 04/06/20**14** | 1050  ***(No)***  ***Greenock*** | WD: 06/06/**14** | | **SU**: hanging |
| **DM**, R  *HMP Greenock*  **[Section 6.1.c]** | 22 | 19/03/20**11** | 16/01/20**13** | 669 | **WD:** 28/02/**13** | | **SU**: hanging |
| **LD**, R  *HMP Perth* | 33 | 02/04/20**11** | 19/11/20**13** | 962  *(Yes)* | **WD:** 05/03/**14** | | **SU**: hanging |
| **ST**, R  *HMP Perth*  **[Section 6.1.e]** | 36 | 27/12/20**11** | 02/12/20**14** | 1071  *(Yes)* | **WD:** 26/02/**15** | | **SU**: hanging |
| **21 deaths in Scottish prison custody in 2012.**  **FIVE out of 21 Fatal Accident Inquiries (FAIs) into prisoner-deaths had time from prisoner-death to FAI-end > 1095 days.**  **In TWELVE out of 13 FAIs with time from prisoner-death to FAI-end > 730 days, the determination could not be located on Scottish Courts & Tribunals website, 17 June 2017.** | | | | | | | |
| **KF**, C  *HMP Edinburgh* | 47 | 08/09/**2012** | 25/06/**2015** | 1020  *(Yes)* | WD:25/06/**2015** | | **UO**: coronary atherosclerosis & nefopam toxicity |
| **RJB**, C  *HMP Glenochil* | 38 | 07/01/**2012** | 20/05/**2013** | 499 |  | | **NC**: metastatic adenoma of right lung |
| **EHAMcD**, C  *HMP Shotts* | 43 | 18/06/**2012** | 12/08/**2014** | 785  ***(No)***  ***Shotts*** | FF | | **NC**: metastatic colon cancer |
| **WS**, C  *HMP Edinburgh* | 81 | 30/06/**2012** | 21/08/**2013** | 417 |  | | **NC**: 1) small bowel infarction, 2) superior mesenteric artery occlusion, 3) atherosclerosis, 4) diabetes mellitus & gout. |
| **EG**, C  *HMP Edinburgh* | 61 | 25/12/**2012** | 25/08/**2014** | 608 |  | | **NC**: 1) ischaemic heart disease, 2) coronary artery atheroma |
| **WDSE**, C  *HMP Glenochil* | 53 | 19/09/**2012** | 08/10/**2013** | 384 |  | | **NC**: 1) cardiomegaly & atherosclerotic coronary artery disease, 2) hepatic steatosis, type 2 diabetes mellitus. |
| **WC**, C  *HMP Glenochil* | 46 | 24/03/**2012** | 20/05/**2015** | 1152  ***(No)***  ***Glenochil*** | WD:20/05/**2015** | | **NC**: 1a) ischaemic heart disease & 1b) atherosclerotic coronary artery disease & 2) liver cirrhosis, chronic drug misuse. |
| **ECB**, C  *HMP Perth* | 76 | 09/05/**2012** | 24/11/**2014** | 929  ***(No)***  ***Open Estate*** | FF | | **NC**: 1) metastatic prostate cancer & its complications 2) hypertensive cardiovascular disease. |
| **JK**, C  *HMP Perth* | 50 | 13/08/**2012** | 24/11/**2014** | 833  ***(No)***  ***Open Estate*** | FF | | **NC**: atherosclerotic coronary artery disease |
| **JCMcC**, R  *HMP Perth* | 44 | 07/09/**2012** | 07/09/**2015** | 1095  ***(No)***  ***Perth*** | WD:07/09/20**15** | | **NC**: a) candida pneumonia & septicaemia, b) chronic obstructive pulmonary disease, c) HIV. Other significant conditions contributing to death but not related to the diseases or conditions causing it are liver cirrhosis & chronic HCV. |
| **SW**, C  *HMP Perth* | 42 | 20/02/**2012** | 26/03/**2014** | 765  ***(No)***  ***Perth*** | WD:31/03/**2014** | | **NC**: acute subdural haemorrhage |
| **PI**, C  *HMP Peterhead* | 50 | 01/01/**2012** | 12/06/**2012** | 163 | WD:13/06/**2012** | | **NC**: coronary artery thrombosis |
| **BP**, C  *HMP Barlinnie* | 42 | 11/03/**2012** | 19/12/**2013** | 648 |  | | **NC**: myocardial infarction |
| **JD**, C  *HMP Barlinnie* | 40 | 17/06/**2012** | 16/10/**2014** | 815  ***(No)***  ***Barlinnie*** | WD:16/10/**2014** | | **SU**: death by hanging |
| **RJMcN**, R  *HMP Inverness* | 19 | 13/10/**2012** | 01/10/**2013** | 353 | WD:11/10/**2013** | | **SU**: death by hanging |
| **RL**, C  *HMP Perth* | 32 | 06/03/**2012** | 25/09/**2014** | 933  ***(No)***  ***Perth*** | FF | | **SU**: 1a) cerebral hypoxia, 1b) low-level suspension by the neck from knotted bed-sheet ligature. |
| **SH**, C  *HMP Shotts* | 31 | 08/11/**2012** | 31/07/**2014** | 630 | ?? | | **SU**: death by hanging |
| **IJM**, C  *HMP Shotts* | 41 | 10/02/**2012** | 03/03/**2015** | 1117  ***(No)***  ***Shotts*** | WD:03/03/**2015** | | **SU**: death by hanging |
| **RMcC**, C  *HMYOI Polmont* | 18 | 13/02/**2012** | 07/08/**2015** | 1271  ***(No)***  ***Polmont*** | FF | | **SU**: death by hanging |
| **SM**, C  *HMP Cornton Vale* | 19 | 12/05/**2012** | 23/03/**2016** | 1411  ***(No)***  ***Cornton***  ***Vale*** | WD:23/03/**2016** | | **SU**: death by hanging |
| **BW**, R  *HMP Barlinnie* | 41 | 02/03/**2012** | 20/05/**2014** | 809  ***(No)***  ***Barlinnie*** | WD:31/05/**2014** | | **SU**: death by hanging |
| **24 deaths in Scottish prison custody in 2013.**  **FIVE out of 24 Fatal Accident Inquiries (FAIs) into prisoner-deaths had time from prisoner-death to FAI-end > 1095 days.**  **In FIVE out of 7 FAIs with time from prisoner-death to FAI-end > 730 days, the determination could not be located on Scottish Courts & Tribunals website, 17 June 2017.** | | | | | | | |
| **PWJG**, C  *HMP Shotts* | 35 | 03/06/**2013** | 17/07/**2014** | 409 | WD:29/07/2014 | | **UO**: amitriptyline & chlorpheniramine intoxication |
| **TAMcC**, C  *HMP Edinburgh* | 53 | 21/04/**2013** | 28/11/**2014** | 586 |  | | **NC**: metastatic hepatocellular carcinoma, HCV, HIV |
| **JAC**, C  *HMP Greenock* | 60 | 11/08/**2013** | 15/09/**2015** | 765  *(Yes)* | WD:15/09/2015 | | **NC**: i) multiple organ failure due to ii) liver cirrhosis with disseminated hepatocellular carcinoma due to iii) Hepatitis C virus infection. |
| **MG**, C  *HMP Low Moss* | 52 | 25/09/**2013** | 19/12/**2014** | 450 | WD:10/03/2015 | | **NC**: haemoptysis due to advanced oesophageal cancer with fistula formation |
| **JAS**, C  *HMP Glenochil* | 60 | 27/02/**2013** | 27/09/**2013** | 212 |  | | **NC**: 1) carcinomatosis, 2) metastatic non-small cell lung cancer, 3) ischaemic heart disease & emphysema |
| **AT**, C  *HMP Edinburgh* | 40 | 04/05/**2013** | 30/06/**2014** | 421 |  | | NC: sepsis due to or as consequence of cirrhosis secondary to HCV & alcohol |
| **GWG**, C  *HMP Glenochil* | 62 | 09/05/**2013** | 07/11/**2014** | 547 | FF | | NC: death due to acute exacerbation of chronic obstructive airways disease |
| **FC**, C  *HMP Addiewell* | 42 | 31/05/**2013** | 28/10/**2016** | 1246  ***(No)***  ***Addiewell*** | WD:16/02/2017 | | NC: multi-drug toxicity |
| **DA**, C  *HMP Glenochil*  **[Section 6.1.c]** | 44 | 15/09/**2013** | 30/04/**2017** | 1323+  ***(No)***  ***Glenochil*** | WD:01/09/2017  Posted:  13/09/2017 | | NC: coronary artery atherosclerosis |
| **HD**, C  *HMP Barlinnie* | 60 | 22/09/**2013** | 02/06/**2014** | 253 | WD:21/08/2014 | | **NC**: 1) ischaemic heart disease; 2) coronary artery atheroma |
| **MNH**, R  *HMP Barlinnie* | 48 | 27/09/**2013** | 28/07/**2014** | 304 | WD:04/08/2014 | | **NC**: intra-abdominal haemorrhage due to a ruptured pancreatic pseudo cyst with splenic vein fistula |
| **GMcK**, C  *HMP Low Moss* | 48 | 25/10/**2013** | 29/07/**2014** | 277 | WD:04/08/2014 | | **NC**: subarachnoid & intracerebral haemorrhage due to berry aneurysm |
| **WKM,** C  *HMP Edinburgh* | 44 | 03/11/**2013** | 18/11/**2014** | 380 | ??:21/01/2015 | | **NC**: coronary atherosclerosis |
| **RT**, C  *HMP Edinburgh* | 65 | 30/11/**2013** | 29/04/**2015** | 515 | WD:09/05/2015 | | **NC**: a) clinically diagnosed multi-organ failure, 1b) complications of cellulitis of lower limbs |
| **RM**, C  *HMP Barlinnie* | 74 | 11/12/**2013** | 26/05/**2015** | 531 |  | | **NC**: 1) bronchopneumonia, 2) cancer of larynx, , ischaemic heart disease & acute limb ischaemia |
| **JW**, C  *HMP Addiewell* | 45 | 25/02/**2013** | 12/08/**2013** | 168 | FF | | **NC**: coronary artery atheroma |
| **JF**, C  *HMP Open Estate* | 25 | 22/05/**2013** | 10/04/**2014** | 323 | FF | | **SU:** death by hanging |
| **DRT**, R  *HMP Barlinnie* | 45 | 14/06/**2013** | 20/10/**2014** | 493 | FF | | **SU:** death by hanging |
| **RWM**, C  *HMP Barlinnie* | 56 | 21/12/**2013** | 12/01/**2015** | 387 | WD:14/01/2015 | | **SU:** 1a) hypoxic ischaemic encephalopathy due to 1b) hanging |
| **DMJC**, C  *HMP Glenochil* | 55 | 23/01/**2013** | 31/03/**2015** | 797  ***(No)***  ***Glenochil*** | WD:20/05/2015 | | **SU:** death by hanging |
| **DTC**, C  *HMP Barlinnie* | 40 | 27/03/**2013** | 06/02/**2015** | 681 | WD:17/04/2015 | | **SU:** multiple injuries due to a fall from height |
| **JJ**, C  *HMP Perth*  **[Section 6.1c+e]** | 28 | 28/09/**2013** | 29/09/**2016** | 1097  ***(No)***  ***Perth*** | WD awaited  WD:28/07/2017  **Posted:**  **08/09/2017** | | **UO: lethal quantities of buprenorphine, gabapentin and phenazepam.** |
| **JSB**, R  *HMP Addiewell* | 24 | 29/09/**2013** | 03/03/**2017** | 1251  ***(No)***  ***Addiewell*** | WD awaited;  actually  WD:08/05/2017 | | **SU:** death by hanging |
| **JP**, R  *HMP Perth*  **[Section 6.1c+e]** | 24 | 23/01/**2013** | 03/03/**2017** | 1428  *(Yes)* | WD:07/04/2017 | | **SU:** death by hanging |

Four FAIs into suicides or overdose of undetermined intent which resulted in

Section 6.1 (c, d, or e) findings.

| **Initials,**  **status C/R**  **convicted/remand**  *HM Prison: SCTS-posted*  ***if posted*** *on website of Scottish Courts & Tribunals by 17/06/20****17*** | | | Age at  Death | **Dates** | | **Section 6.1 Findings:**  **c), d) or e).** | **Cause of death** |
| --- | --- | --- | --- | --- | --- | --- | --- |
|  |  |  |  | Reception  **Death** | WD:  **Wait**; *Sheriff* |  |  |
| **ST**, R  *HMP Perth: SCTS-posted* | | | 36 | 08/12/2011  **27/12/2011** | 26/02/20**15:**  **1157 days**;  *LDRF* | **Section 6.1.e)** | **SU: hanging from knotted bed-sheet used as ligature tied to upper bunk bed-frame in cell.** |
| *Determination in brief available from author* | | | | | | | |
| **Section 6.1.e)** | | | E1 **Vulnerability assessment form completed by police custody security officers in respect of persons held in police custody should accompany such persons in addition to the Personal Escort Record.**  E2 **Scottish Prison Service to establish an effective system whereby any concerns by persons visiting prisoners as to the wellbeing of prisoners, including concerns of self-harm and suicide, can be brought to the attention of the appropriate prison staff.**  E3 **Said system should include an effective method for making visitors to prison aware of how to bring such concerns for the wellbeing of prisoners to the attention of the appropriate prison staff.** | | | | |
| **DM**, R  *HMP Greenock* | | | 22 | 16/03/2011  19/03/20**11** | 28/02/20**13:**  **712 days**; *TW* | **Section 6.1 c)** | **SU: hanging.** |
| *Determination in brief available from author* | | | | | | | |
| **Section 6.1.c)** | | **Reasonable precaution would have been for X or Y (both named) to ensure the**  **implementation of Rule 94(10), ie: segregated prisoner to be assessed by doctor as soon as practicable & as often as necessary thereafter but at least once a week.**  [Doctors at HMP Greenock worked part-time; Rule 94(10) has since been removed from the Rule Book.] | | | | | |
| **JP**, R  *HMP Perth:*  *SCTS-posted* | | | 24 | 16/01/2013  **Found on afternoon of 19/01/2013; death at**  **Perth Royal Infirmary on 23/01/2013** | 07/04/**2017:**  **1463 days**;  *LDRF* | **Section 6.1 c) & e)** | **SU: ligature pressure on neck from hanging himself by a shoelace tied to top of bed.** |
| *Determination in brief available from author* | | | | | | | |
| **Section 6.1 c)+e)** | | | **Reasonable precaution whereby the death of JP might have been avoided was:**  **C1. PO1 placing a copy of letter from Perth Procurator Fiscal Depute, dated 16^th^ January 2013 addressed to Governor, HMP Perth, in JP’s prison medical records & attaching copy of said letter to the ACT2care booklet re JP which was duly completed on his admission to HMP Perth on 16^th^ January 2013.**  E1 **PO2 & Nurse should have been appraised of the said letter, which constituted additional information as defined in ACT2Care reception risk assessment form, by PO1 before they carried out Reception & Health Care Risk Assessments for JP on 16^th^ January 2013.**  E2 **Personal Escort Record (PER) for JP made no mention of his having been subject to constant observations during part of his period in police custody on 15^th^ and 16^th^ January 2013 and no specific provision on the said form to note that he was subject to constant observations at certain times whilst in police custody.**  E3 **Doctor was not provided with a copy of JP’s PER when he carried out his assessment of JP on 17^th^ January 2013.** | | | | |
| **JJ**, C  *HMP Perth:*  *SCTS-posted on*  *08/09/2017* | | | 28 | 24/09/2013  **28/09/2013** | 28/07/**2017:**  **1399 days**;  *LDRF* | **Section 6.1 c)** **& e)** | **UO: lethal quantities of buprenorphine, gabapentin and phenazepam.** |
| *Determination in brief: further detail available from author* | | | | | | | |
| JJ had a heroin habit of at least 4-years, also took cannabis; and had Attention Deficit Hyperactivity Disorder (ADHD). JJ was sentenced to 45 weeks’ imprisonment. On reception at HMP Perth on 24^th^ September 2013, he did not appear to be under influence of drugs or alcohol, being lucid and coherent, and at no apparent risk of self-harm but PER noted his ADHD. JJ indicated that he only smoked heroin; urine was positive for opiates and cannabis.  Transferred to B Hall and locked in cell B 3/32. Soon thereafter, prisoners congregated at his cell and POs suspected that JJ had banked drugs internally. Therefore, on the evening of 24^th^, JJ was transferred to Separation and Reintegration . . .  Friday 27^th^ September, prior to return to B Hall at 1.45pm, JJ was subject to sniffer dog search which was negative. JJ’s presentation had improved by account of POs who had observed him most. Locked in cell for 45 minutes, then 90 minutes of association with other prisoners. Demeanour unremarkable until at least 4.40pm but by 5.30pm, JJ was incoherent and under influence of some substance. Nurse attended: JJ was oriented to identity, time & place but would not disclose what he had taken. Unsteady on feet & unable to provide urine sample – container left. [Dipstick test could only have revealed opiates or cannabis]. JJ was put under hourly observation requiring verbal & visual response. If condition deteriorated, contact healthcare team. Between 6.40pm & 8.40pm, JJ ‘sleeping most of the time’, was not coherent & did not know PO1 was there who checked on his welfare.  PO2 took over observations from 9pm. From 11pm to 7am, JJ recorded as snoring loudly, in deep sleep (? no verbal responses) and to have moved during the night . . .  Saturday 28^th^ September: At 8am, PO1 & PO2 entered JJ’s cell: JJ was lying on his side, facing middle of cell with head towards door. POs claimed to have obtained verbal response in form of a grunt – disbelieved on basis of autopsy.  PO2’s records re increased sound of JJ’s snoring (latterly very loud and noticeable) accords with medical opinion re effects on someone suffering overdose. Based on PM, unlikely that JJ could speak at 8am, contrary to evidence of POs.  *Overdose death within 1st week after reception. Incorrectly performed and/or misrepresented hourly observations.* | | | | | | | |
| **Section 6.1 c)** **& e)** | **Reasonable precautions whereby death might have been avoided were:**  **C1. had JJ avoided taking non-prescribed drugs illegally and in lethal quantities on his return to B Hall at 1.45pm on 27 September 2013;**  **C2. had JJ, when asked at 5.30pm, told nurse which illegal drugs he had taken;**  **C3. had POs conducting hourly observations on JJ been provided with information about adverse drug effects and how symptoms might exhibit themselves;**  **C4. had POs been informed of the changes in the prisoner’s presentation which might indicate deterioration.**  **C5. that POs, undertaking hourly observations on a prisoner suspected to having taken drugs, ensured that they obtained both a visual and coherent verbal response to confirm that prisoner was oriented to time, place and identity;**  **C6. had PO, having determined that JJ was not oriented to time, place and person, sought a further medical assessment for JJ.**  **Other relevant facts wrt JJ’s death were:**  **E1. That nursing staff called to assess prisoner suspected of taking drugs should carry with them standard equipment to measure accurately blood pressure, pulse, blood sugar, oxygen saturation and breathing;** [Sheriff did not mention carriage by nursing staff of Naloxone (opiate antidote) to be injected intramuscularly]  **E2. That nursing staff called to assess prisoner suspected of taking drugs should have access to Vision system to have up-to-date medical information about the prisoner;**  **E3. If prisoner who is suspected of taking drugs is unable to provide urine sample, a procedure should be set up to ensure that such a sample is provided by the prisoner when he is able to do so.** | | | | | | |

Three FAIs into deaths from natural causes in 2010-2013 which resulted in

Section 6.1 (c, d or e) findings; together with account of FAI into AH’s death in October 2014

| **Initials,**  **status C/R**  **convicted/remand**  *HM Prison: SCTS-posted*  ***if posted*** *on website of Scottish Courts & Tribunals by 17/06/20****17*** | Age at  Death | | **Dates** | | **Section 6.1 Findings:**  **c), d) or e)** | **Cause of death** |
| --- | --- | --- | --- | --- | --- | --- |
|  |  |  | Reception  **Death** | WD:  **Wait**; *Sheriff* |  |  |
| **GJC**, C  *HMP Glenochil* | 78 | | As transfer from HMP Peterhead 03/12/20**10**  **07/03/2011** | 07/07/20**14:**  **1218 days**;  *DO’C* | **Section 6.1.e)** | **NC: intracerebellar haemorrhage, secondary to long-standing hypertension. Less than 24 hours before death, a metastatic non-small cell lung cancer was diagnosed which had spread to liver but said cancer did not materially contribute to cause of death – contrary to original autopsy.** |
| *Determination in brief: further detail available from author* | | | | | | |
| Sheriff noted with concern the following procedural aspects of FAI for GJC. Sheriff’s timely but time-pressured preparation had fortunately led to his questioning, and rejecting, an agreed **joint minute**:   a) Delay in commencement of FAI as petition for FAI was not lodged until 2-years after GJC’s death, which risked loss of evidence, see Cullen Review at paragraphs 6.14 and 6.22. b) Evidence was lost - destruction of autopsy samples (then typical after 2 years) took place shortly before Dr S went to look for them even though it was known that FAI is mandatory for prisoner-deaths. Samples are not kept indefinitely where autopsies involve 2 pathologists unless marked for non-disposal. c) Initial approach by Crown (and SPS) re procedural matters on 31 July 2013: on 30 July 2013, Sheriff viewed for the first time a large number of documents on encrypted e-drive lodged by the Crown in process, including unsigned statements, copy medical records, **a joint minute**, medical expert review by Dr G and the first autopsy report. Joint minute included agreement between Crown and SPS on many matters including that cause of death was “complications of metastatic carcinoma” and a very abbreviated account of GJC’s medical attention between July 2010 and March 2011. Sheriff was told that circumstances of death were uncontroversial, that no oral evidence need be led and that unsigned statements, incorporated into the joint minute, would suffice as evidence. Fortunately, Sheriff had had time to read some of these documents before the preliminary hearing and expressed surprise that Crown proposed to proceed in this way because i) Rules do not permit evidence to be given in unsigned statements, ii) there were contradictions, not least between cause of death at autopsy and by expert medical review. | | | | | | |
| **Section 6.1. e)** | E1 **GJC suffered from cancer of lung with liver metastasis from at least September 2010 from which he showed non-specific symptoms and remained undiagnosed/untreated until his ICH death.**  E2 **Failure to diagnose arose from a series of shortcomings, both personal and institutional, at Aberdeen Royal Infirmary, HMP Peterhead and HMP Glenochil (detailed at notes 131-137).**  E3 **But for these shortcomings, cancer would have been diagnosed earlier, possibly before end of 2010.**  E4 **Absence of timely diagnosis meant that GJC missed out on i) palliative treatment which might have improved his quality of life; ii) chance of being considered for compassionate release.**  E5 **Diagnostic failure did not, however, have an effect on timing of ICH from which GC died.**  *FIVE recommendations were made to remedy the above shortcomings:*   1. *ARI Radiology Department to ensure there are clear and uniform protocols for handling imaging requests that are not unconditionally accepted – ideally by discussion with requesting physician to avoid unnecessary delays.* 2. *Those making requests should have in place systems to monitor the outcome of every request & timeously identify any request which has not been carried out.* 3. *Prisoner records should contain an accurate, comprehensive and regularly updated, easily accessible summary of the patients’ significant medical history and current issues to enable the prisoner’s appropriate treatment.* 4. *Review and standardize procedures for prisoner transfers to ensure that a careful review is made of the prisoner’s medical position by the transferring prison so that all notable features, especially in relation to current treatment and investigation, are clearly brought out in the prisoner’s medical records.* 5. *Review & standardize procedures in the receiving prison to ensure that, within a short period of transfer, doctor consults with prisoner & reviews his/her medical position with reference to his/her medical records & any observations made by other healthcare staff following reception.* | | | | | |
| **NFMcN**, C  *HMP Glenochil* | 72 | | As transfer from HMP Peterhead  04/11/2010  **31/07/2011** | 16/02/20**14:**  **931 days**;  *DNM* | **Section 6.1.e)** | **NC: metastatic large B cell lymphoma of colon, sepsis; secondary causes of atherosclerotic coronary vascular disease & hypertension.** |
| *Determination in brief available from author* | | | | | | |
| **Section 6.1 e)** | **Other relevant factors were: lack of communication between doctors & lack of continuity of care was evidenced; together with delays and inefficiencies re arrangements for blood tests and other diagnostic investigations outside of the prison (eg pre-handover to NHS Scotland, with prison doctors having no access to Scottish Care Information Store).** *Prison now works with a portfolio of regular GPs rather than via an agency & its doctors are connected to electronic database which gives them the same facility to access diagnostic test & scan results as other practitioners.* | | | | | |
| **DA**, C  *HMP Glenochil*  *SCTS-posted* | 44 | | 20/01/2011 into custody  **15/09/2013** | 01/09/**2017:**  **1447 days**;  *SGC* | **Section 6.1. c)** | **NC: coronary artery atherosclerosis** |
| *Determination in brief* | | | | | | |
| DA was smoker; Body Mass Index of 32; suffered from sciatica; received prescriptions of omeprazole (antacid), amoxicillin (antibiotic), mirtazapine (antidepressant) together with co-codamol and nefopam (analgesics). No known cardiac conditions.  3 August: at 09.50am, while working in prison garden DA complained of chest pain with associated sweating and shortness of breath; nurse attended, pain resolved, rest advised & contact healthcare centre if pain returned. Overnight, non-acute pains on left side of chest & shoulder.  24 August: crushing pains across chest & left arm felt cold & numb – nurse attended, DA had high BP and fast pulse, 300mg aspirin and hospitalized for further assessment (09.50hrs) at A&E of Forth Valley Royal Hospital in Larbert (FVRHL), where ECG, troponin (normal @ <50 and so not indicative of recent cardiac arrest). Admitted for repeat troponin test & ECG (only slightly inverted T waves) - neither of concern.  25 August: Exercise Tolerance Test (ETT) booked for DA who was then discharged . . .  15 September: DA activated cell alarm at 01.16hrs on account of chest pains, chill in left arm & feeling unwell. Two POs attended and spoke to DA thro spyhole. DA was lucid & not ashen, nor short of breath. PO1 phoned 1^st^-line manager (M) who attended at 01.30hrs with two other POs. M and PO2 entered DA’s cell who was sitting fully clothed in his chair. DA recounted hospitalization and that he’d been told he might have had a minor heart attack. Chest pain had been 6 on scale of 1-10 but had subsided & DA was beginning to feel better. M told DA that M would phone on-call Dr. to get further medical advice. M phoned Dr. B at 01.40hrs – DA was already known to Dr. B who was aware of DA’s test results from FVRHL. As symptoms were subsiding, Dr. B did not consider emergency ambulance call-out but sufficiently concerned to go to prison himself to see DA.  M returned to DA’s cell at 01.55 hrs. Cell door was closed, POs 1+2 were still in attendance and the two other POs also in vicinity. M looked thro’ spyhole & saw DA sitting in his chair with head back. M & PO1 entered cell, spoke to DA but got no response – he was unconscious, officers did not attempt to detect pulse because they thought DA still breathing as he was “making gurgling, breathing like sounds”. In fact, DA was in ventricular fibrillation (VF) and had entered this condition when he lost consciousness: the sounds were agonal breathing. DA’s age and condition were consistent with his breathing agonally for several minutes.  Treatment of VF is primarily by a combination of cardiopulmonary resuscitation (CPR) and defibrillation . . . On night of 14-15 September, no defibrillators or Automated External Defibrillators (AEDs) were readily accessible at HMP Glenochil – there was a defibrillator in locked heath centre. Only PO1 even knew about this location. Unclear also whether “crash pack” included resusciades.  M contacted control room to call for ambulance at 01.56hrs & phoned Dr B to abort his journey as ambulance en route. POs 1+2 thought that DA had stopped breathing at 01.58 & radioed code blue but did not take pulse. Ambulance control received call at 01.57, ambulance en route by 01.58, arrived at prison gate at 02.01, parked at DA’s hall by 02.04 and reached DA’s cell at by 02.06 at latest. On arrival of paramedic, DA was cyanosed, lying on his back in cell, one PO in doorway, others outside cell & no-none performing CPR. Defibrillator displayed 02.05, DA shocked by 02.06 but remained in VF. Paramedic instructed POs to perform CPR while she intubated & canulated DA for him to receive adrenalin & amiodarone. Further shocks & adrenalin plus ongoing CPR without success. DA never regained consciousness, died in ambulance & pronounced dead shortly after arrival at FVRHL at 03.32hrs.  At PM, DA had fractures consistent with CPR. No gross abnormality of myocardium but there was coronary artery atherosclerosis within left anterior descending and circumflex arteries – severe stenosis of vessel lumen so that cross-sectional diameter was reduced by 70%. Likely that DA had a thrombosis caused by his coronary artery disease. Microscopic examination also revealed small area of damage to DA’s heart where there had been a previous episode of ischaemia.  Five AEDs now available at HMP Glenochil, one in each hall, health centre and main reception area. A sixth was later installed in main link corridor – so that AED can be brought to any cell within 5 minutes.  **Joint-minute** had been lodged and sheriff was invited to make Formal Findings only, which he declined to do and asked Crown to give consideration to identifying and calling further witnesses (including paramedic who attended DA). On 19 January 2017, inter alia including a second joint-minute, attention was drawn to WD of 29 September 2016 following FAI into death of AH at HMP Dumfries, a case with similarities to that of DA, **please see AH below.**  **CCTV evidence – or the lack of it.**  Following DA’s death, the Crown had failed to request from SPS the CCTV footage with time-stamps of comings and goings outside of DA’s cell; and the footage had since been destroyed - rather than submitted by SPS – because the evidence had not been requested. Sheriff was made aware very late on about this failure to have secured evidence which would have resolved factual disputes and uncertainties of timing/actions taken in respect of CPR by POs and when, exactly, did M and PO1 find DA unconscious in his cell, apparently at 01.50hrs but control room was contacted by radio only at 01.56hrs. Sheriff concludes that DA was found collapsed at nearer 01.55hrs. Sheriff also found it extraordinary that Crown had initially intended to lead no evidence about the presence or absence of defibrillators within HMP Glenochil, let alone training in their use. Despite PO’s evidence that availability of defibrillator might have made a difference to DA, Crown & SPS had still moved to close the inquiry with only Formal Findings – instead Sheriff had asked for expert medical opinion (from cardiologist).  Sheriff also questioned that prisoners had equivalent medical cover at night as other citizens because others can take themselves to hospital whereas prisoners cannot but must await ambulance to be called on their behalf. | | | | | | |
| **Section 6.1. c)** | **Reasonable precautions whereby death might have been avoided were:**  **C1. Automated external defibrillators (AED) could have been made readily accessible for use by prison officers on night shift at HMP Glenochil;**  **C2. Prison officers on night shift at HMP Glenochil could have been given training and instruction in relation to when and how to use an AED.** | | | | | |
| ***AH****, C*  *HMP Dumfries:*  *SCTS-posted* | *27* | | *30/04/2014*  ***03/10/2014*** | *29/09/****2016:***  ***727 days****;*  *BAM* | ***Sections 6.1. c), d) & e)*** | ***NC****:* ***Ischaemic heart disease due to coronary artery atheroma.*** |
| *Determination in brief* | | | | | | |
| ***AH, who had a diagnosed history of diabetes mellitus (DM) type 1 and administered his own insulin, was in a double-occupancy cell with YY.*** *By 3 October 2014, the two had cell-shared for about one month. DM is risk factor for coronary artery atheroma. AH also received Ranitidine daily for heartburn, took anti-depressant medication and pain relief for recurrent shoulder pain.*  ***On afternoon of 3 October 2014, AH complained to Prison Officer (PO) that he had chest pains.*** *As they persisted at 5pm, PO contacted prison medical centre & arranged for AH to be seen there. AH told nurses that he had central chest pain which had lasted for three to four hours, a “twisting or grabbing” type of pain with no radiation to his back or arms. He thought it was heartburn as this was a longstanding condition:* ***AH*** ***had taken two Ranitidine - his prescribed heartburn pills - earlier in the day, but his heartburn had not eased.***  *Pulse & blood pressure were within normal range.* ***Nurses decided to do ECG before consulting doctor who was off-site. Unable to perform ECG – machine functioning but no paper to fax printed-out result to doctor. Dr, who had no access to AH’s records, advised nurses to monitor & he (ie doctor) could see AH at morning clinic.***  *Nurse noticed that AH had not returned for his prescribed night medication. She arranged for him to be brought back to medical centre: AH was still suffering from chest pain which eased when he sat upright. He complained also of a throbbing pain in his shoulder and was noted to have a crackly cough. Appeared brighter than at the time of his earlier visit and in good spirits. His blood pressure was taken again and remained within normal range. Nurses formed the view that his symptoms were improving but told AH that if he felt unwell during the night, he should let someone know. He was returned to cell for the night.*  ***Shortly before 11pm, cellmate was alerted to AH’s banging on his bed from below and groaning in pain.*** *JD spoke to AH and concluded that he was in severe distress. His breathing was shallow; and he was unable to speak. JD decided to press the emergency cell button to seek assistance shortly after 11pm.*  *Prison protocol required a team of three to be present before a cell could be opened.  Officer who attended JD’s call alerted colleagues using a ‘Code Blue’. Cell opened 11.04pm; CPR attempted; paramedics arrived with defibrillator at 11.22pm. Taken to hospital, where AH was pronounced dead at 11.55pm. Autopsy found changes indicative of acute (+ signs of past) infarction.* | | | | | | |
| ***Section 6.1. c), d) & e)*** | | *C1* ***For nurses to have completed an electrocardiogram on AH.***  *C2* ***For prison doctor to have advised nurses to take further action if AH’s presenting symptoms continued into the evening.***  *C3* ***For nursing staff to have called an ambulance for AH at around 8pm on 3 October 2014.***  *D1* ***Electrocardiograph (ECG) machine in the medical centre at HMP Dumfries on 3 October 2014 was not functioning correctly. Machine had no paper to print the ECG results, and nursing staff had no access to replacement paper for it. Printing was necessary because the nurses were not trained to interpret the ECG test results and needed to fax printed results to a doctor’s surgery outside the prison.***  *E1* ***Defibrillator machines to be available at HMP Dumfries for emergency use when prison medical centre is closed.***  *E2* ***SPS to install & maintain a system of individual cell emergency alarms at all times.***  *E3* ***SPS to consider enabling prison doctor to access relevant prisoner medical records when giving advice from outside of the prison.*** | | | | |
